# Increased viperin expression induced by avian infectious bronchitis virus inhibits viral replication by restricting cholesterol synthesis: an in vitro study

**DOI:** 10.1186/s13567-024-01368-w

**Published:** 2024-09-27

**Authors:** Yu Zhang, Tao-Ni Zhang, Yan-Peng Lu, Li-Na Ren, Sheng-Ting Chen, Ling Liu, Lan-Ping Wei, Ji-Ming Chen, Jian-Ni Huang, Mei-Lan Mo

**Affiliations:** 1 College of Animal Science and Technology, Guangxi University, Nanning, 530004 China; 2 Guangxi Zhuang Autonomous Region Engineering Research Center of Veterinary Biologics, Nanning, 530004 China; 3 Guangxi Key Laboratory of Animal Reproduction, Breeding and Disease Control, Nanning, 530004 China

**Keywords:** Infectious bronchitis virus, viperin, cholesterol

## Abstract

With the emergence of new variant strains resulting from high mutation rates and genome recombination, avian infectious bronchitis virus (IBV) has caused significant economic losses to the poultry industry worldwide. Little is known about the underlying mechanisms of IBV-host interactions, particularly how IBV utilizes host metabolic pathways for efficient viral replication and transmission. In the present study, the effects of the cell membrane, viral envelope membrane, and viperin-mediated cholesterol synthesis on IBV replication were explored. Our results revealed significant increase in cholesterol levels and the expression of viperin after IBV infection. Acute cholesterol depletion in the cell membrane and viral envelope membrane by treating cells with methyl-β-cyclodextrin (MβCD) obviously inhibited IBV replication; thereafter, replenishment of the cell membrane with cholesterol successfully restored viral replication, and direct addition of exogenous cholesterol to the cell membrane significantly promoted IBV infection during the early stages of infection. In addition, overexpression of viperin effectively suppressed cholesterol synthesis, as well as IBV replication, whereas knockdown of viperin (gene silencing with siRNA targeting viperin, siViperin) significantly increased IBV replication and cholesterol levels, whereas supplementation with exogenous cholesterol to viperin-transfected cells markedly restored viral replication. In conclusion, the increase in viperin induced by IBV infection plays an important role in IBV replication by affecting cholesterol production, providing a theoretical basis for understanding the pathogenesis of IBV and discovering new potential antiviral targets.

## Introduction

Avian infectious bronchitis virus (IBV) is a highly contagious and deadly coronavirus that can cause devastating upper respiratory tract disease in chickens. Infectious bronchitis (IB) caused by IBV has caused substantial economic loss in the poultry industry and affects meat quality, egg production, and the reproductive system [1–3]. To date, vaccination remains the main effective measure to prevent and control this disease. Nevertheless, high mutation rates and genetic recombination have led to the frequent emergence of new variants, which seriously affects the efficiency of existing vaccines [4–6]. Therefore, novel drugs or therapeutic agents to control viral spread and disease progression are urgently needed. Further in-depth exploration of the potential mechanisms of virus-host-cell component interactions is important for developing effective vaccines and therapeutic drugs against IBV.

Enveloped viruses must cross the envelope membrane during their infection cycle of entry and budding from the host cell. Thus, the nature and integrity of the membrane are important determinants of effective infection [7]. For the enveloped virus to successfully enter the cell, it needs to bind to specific cell receptors and fuse the viral membrane with the cell membrane [8]. There is increasing evidence that the entry of enveloped viruses may require both the cell membrane and the viral envelope membrane or that both membranes contain cholesterol [7, 9, 10]. The lipid raft is a special membrane microregion that is rich in cholesterol, sphingolipid, and related proteins and is an important part of the cell membrane [11–13]. Cholesterol has been shown to be critical to the viability of several coronaviruses, which enter cells from cholesterol-rich lipid rafts, such as severe acute respiratory syndrome coronavirus 2 (SARS-CoV-2) [12, 14–16], severe acute respiratory syndrome virus (SARS-CoV) [17], porcine epidemic diarrhea virus (PEDV) [18], and mouse hepatitis virus (MHV) [19]. Thus, cholesterol depletion disrupts the integrity of cell membranes or viral envelope membrane lipid rafts, further affecting viral replication. Methyl-β-cyclodextrin (MβCD) is a cholesterol-binding agent used to remove cholesterol and destroy lipid rafts. Multiple studies have shown that MβCD can be used to disrupt canine coronavirus (CCoV) [9], porcine delta coronavirus (PDCoV) [20], and type 1 feline coronavirus (FCoV) [21] cholesterol-rich lipid rafts in the envelope and host membrane, leading to a dose-dependent reduction in viral infection. Despite the enormous progress in IBV research in the last decade, the major virus-host interactions that regulate IBV replication and propagation remain poorly understood. Previous studies reported that lipid rafts and low pH are indeed necessary for IBV entry and that IBV enters cells mainly through clathrin-mediated endocytosis (CME) [22, 23]. Wang et al. [23] demonstrated that MβCD inhibits the IBV entry step but not the post-entry process of the virus, suggesting that a complete lipid raft is required for IBV entry. Another study revealed that IBV structural proteins are colocalized with lipid rafts on the plasma membrane and that the mevastatin- and MβCD-induced destruction of Vero cell lipid rafts inhibits IBV infection, suggesting that lipid rafts on the cell surface may mediate viral adhesion and thus promote IBV endocytosis [24]. Currently, relatively few studies on the effects of cholesterol on IBV replication exist, and they have focused mainly on cholesterol in the cell membrane and have not addressed cholesterol in the viral envelope membrane.

Host cell cholesterol is a key factor in the replication and spread of enveloped viruses. It is widely known that viral assembly, outgrowth, and replication are highly dependent on cellular cholesterol levels, suggesting the importance of host factors in viral infection [7, 10, 25]. Viperin (also known as RSAD2, viral inhibitory protein, endoplasmic reticulum-associated, interferon-inducible) is an antiviral host protein localized to the endoplasmic reticulum and lipid droplets that exhibits broad-spectrum antiviral activity [26–28]. In recent years, viperin has emerged as an important player in the maintenance of cellular cholesterol homeostasis. Various previous studies have linked viperin to the downregulation of cholesterol biosynthesis. Viperin expression inhibits influenza A virus (IAV) replication through binding and inhibition of farnesyl diphosphate synthase (FPPS) synthesis, which in turn interferes with its release from the plasma membrane [29]. Another study showed that the ability of viperin to inhibit rabies virus (RABV) in RAW264.7 cells is achieved by lowering cholesterol and sphingomyelin on the cell membrane and that Toll-like receptor 4 (TLR4) inhibitors inhibited viperin expression in RABV-infected RAW264.7 cells [30]. A recent study revealed that transient expression of viperin in HEK293T cells reduced cholesterol levels by 20 to 30% and identified several cholesterol biosynthetic enzymes, including lanosterol synthetase (LS) and squalene monooxygenase (SM) [31]. However, at present, there are no studies on the inhibition of IBV replication by viperin or the effect of viperin on cholesterol synthesis and thus IBV replication.

In this study, we investigated the effects of the cell membrane and viral envelope membrane on IBV replication, and the results suggested that intact cholesterol on the cell membrane and viral envelope is essential for the effective invasion of IBV. We also examined the antiviral role of viperin and demonstrated that viperin plays a negative role during IBV infection. Moreover, we further explored the effect of viperin-mediated cholesterol synthesis on IBV replication. We found that the inhibitory effect of viperin on IBV could be reversed through exogenous replenishment of host cell membrane cholesterol. To our knowledge, this study is the first to report that viperin inhibits cholesterol synthesis and thus IBV replication, providing a theoretical basis for a deeper understanding of the pathogenic mechanisms of IBV and the identification of new potential antiviral targets.

## Materials and methods

### Cells and virus

Vero and chicken HD11 cells were maintained in our laboratory and cultured in Dulbecco’s modified Eagle’s medium (DMEM, Procell, Wuhan, China) supplemented with 10% fetal bovine serum (FBS, Procell, Wuhan, China) and 1% penicillin, streptomycin and gentamicin (Solarbio, Beijing, China) under 5% CO_2_ at 37 °C. The IBV Beaudette strain (GenBank: DQ001339) was kindly donated by Prof. Ding-Xiang Liu of South China Agricultural University.

### Cellular cholesterol concentration assay and filipin staining of IBV-infected Vero cells

Vero cells were seeded into 12-well plates and then infected with IBV at a multiplicity of infection (MOI) of 1.0 for 12, 24, 36, or 48 h. After that, the cells were washed thrice with sterile phosphate-buffered saline (PBS) and incubated in PBS containing 2% Triton X-100 (Biosharp, Hefei, China) at 37 °C for 30 min, and the supernatant was then collected by centrifugation. The concentrations of cholesterol in the harvested cells were assayed by the Amplex^®^ Red Cholesterol Assay Kit (Invitrogen, Carlsbad, USA) following the manufacturer’s directions. Cholesterol accumulation was determined using filipin III dye (Cayman, Ann Arbor, USA). IBV-infected cells were fixed with 4% paraformaldehyde at room temperature for 15 min and then permeabilized with the permeable solution at room temperature for 10 min, followed by blocking with 5% bovine serum albumin (BSA) for 45 min and adding filipin III dye (working solution with a final concentration of 50 µg/mL with 0.01 mol/L PBS) for 2 h. Then, propidium iodide (PI; Solarbio, Beijing, China) working solution with a final concentration of 20 µg/mL was prepared with 0.01 mol/L PBS, and the cells were incubated with the nuclear dye PI for 15 min. After washing three times with PBS, fluctuations in cholesterol were observed via fluorescence microscopy.

### Western blotting analysis of viperin and IBV N protein expression

Vero cells were harvested and lysed with RIPA lysis buffer (Beyotime, Beijing, China). The samples were then resolved by SDS-PAGE and transferred to PVDF membranes to identify protein expression. The expression of viperin was analysed using a mouse anti-GFP monoclonal antibody (MAb) and a mouse anti-*β*-tubulin MAb (Abmart, Shanghai, China; diluted 1:2000) as the primary antibodies. A specific rabbit polyclonal antibody against the IBV N protein (prepared in our laboratory, diluted 1:1000) was used to detect the expression of the N protein. The signal was further detected by using horseradish peroxidase-conjugated goat anti-mouse IgG or goat anti-rabbit IgG secondary antibodies (Abmart, Shanghai, China; diluted 1:5000).

### qRT-PCR detection of IBV, viperin, and other antiviral genes

Vero and HD11 cell samples subjected to different treatments were collected, and total RNA was extracted from treated cells via an RNA isolator total RNA extraction reagent kit (Vazyme, Nanjing, China) and reverse transcribed into cDNA using the HiScript^®^ III All-in-one RT SuperMix Perfect for qPCR Kit (Vazyme, Nanjing, China) according to the manufacturer’s instructions. The expression levels of the IBV N gene [32] and the viperin gene [33] were determined by absolute qRT-PCR, which was also used to evaluate the efficiency of viperin transfection and the degree of viral replication. Absolute quantitative mRNA levels were calculated using standard curves. The relative mRNA expression levels of IFIT5, IRF7, MX1, NLRC5, OASL, RSAD2, and TLR3 [32–34] are presented as fold changes and were calculated via the 2^−ΔΔCT^ method [35]. The primers used are shown in Table 1, and the relative expression was calculated according to the endogenous control (GAPDH) [32]. The data show changes in multiples of the control.

**Table 1:** **The primers used for qRT-PCR**

Gene name	Sequence (5′-3′)	Amplicon size (bp)	Accession number	Reference
N	F: CAGAAGAAGGGCTCTCGCATTAC R: AGGTTGAGCATTGCCGTAACAC	200	FJ548847	[32]
Viperin/ RSAD2	F: CAGTGGTGCCGAGATTATGCR R: CACAGGATTGAGTGCCTTGA	105	NM_001318443.1	[33]
IFIT5	F: CTCCCAAATCCCTCTCAACA R: AAGCAAACCCACAATCATCA	167	NM_001320422.2	[33]
IRF7	F: ACACTCCCACAGACAGTACTGA R: TGTGTGTGCCCACAGGGTTG	134	NM_205372.2	[32]
MX1	F: GAGCCTTCAGCTGTAGAACCCAAA R: GCCAGCATACACAACCAGAGCAAA	129	GQ390353.1	[34]
NLRC5	F: ACGCAAGTGACCAGTCCTCT R: TGAGTCCAGGCGTTTCTGTT	101	NM_001318435.2	[34]
OASL	F: CACGGCCTCTTCTACGACA R: TGGGCCATACGGTGTAGACT	109	NM_001397447.1	[33]
TLR3	F: GCAACACTTCATTGAATAGCCTTGAT R: GCCAAACAGATTTCCAATTGCATGT	92	EF137861.1	[32]
GAPDH	F: GGTGGTGCTAAGCGTGTTA R: CCCTCCACAATGCCAA	179	X01578.1	[32]

F: forward, R: reverse, IFIT5: interferon-induced protein with tetratricopeptide repeats 5, IRF7: interferon regulatory factor 7, MX1: MX dynamin-like GTPase 1, NLRC5: NLR family CARD domain containing 5, OASL: 2’-5’-oligoadenylate synthetase like, RSAD2: radical S-adenosyl methionine domain containing 2, TLR3: toll-like receptor 3.

### TCID_50_ assay of IBV titres

Vero cells were seeded in 48-well plates and then infected with serial 10-fold dilutions (from 10^1^ to 10^8^ dilutions) of IBV samples in six replicates at 37 °C for 2 h; after washing with sterile PBS, 0.2 mL of DMEM containing 2% FBS was added to each well, and the cells were continually cultured at 37 °C for 72 to 96 h, followed by counting the cytopathic effect wells of each dilution of IBV. Viral titres (TCID_50_) were calculated via the Reed‒Muench method [36].

### Analysis of the effects of depletion and supplementation with cell membrane cholesterol on IBV entry in Vero cells

MβCD (Solarbio, Beijing, China), a cholesterol-depleting drug, was used to deplete cholesterol from the cellular and viral envelope membranes. To investigate the effect of cholesterol depletion on virus entry, Vero cells were pretreated with different concentrations of MβCD (2.5, 5, and 10 mM/mL) for 1 h at 37 °C. Thereafter, the Vero cells were washed with PBS to remove the drug and infected with IBV (MOI = 1.0) for 2 h at 37 °C; after the liquid was removed, the cells were supplemented with 2% maintenance culture solution for an additional 48 h, after which IBV replication capability was determined.

For cholesterol supplementation experiments, Vero cells were pretreated with 10 mM/mL MβCD for 1 h at 37 °C as described above. After washing with PBS, various concentrations of cholesterol (Sigma‒Aldrich, St. Louis, MO, USA) (0.25, 0.5, and 1.0 mg/mL) were added to Vero cells for 1 h, followed by incubation with IBV (MOI = 1.0) for 2 h. Cell samples were collected at 48 h post-infection (hpi) as described above, and viral replication was tested. In addition, the cells were directly treated with different concentrations of cholesterol for 1 h and incubated with IBV (MOI = 1.0) for 2 h. Then, the cells were harvested at 12 and 24 hpi, and the level of IBV replication was determined.

### Analysis of the effects of the depletion and replenishment of cholesterol in the viral envelope membrane on IBV replication in Vero cells

To investigate the functional role of cholesterol in the viral envelope in IBV replication, experimental depletion of cholesterol with MβCD was carried out in the viral mixture. For cholesterol extraction, IBV was incubated with MβCD at concentrations of 2.5, 5, and 10 mM/mL for 1 h at 37 ℃. Vero cells were then infected with a mixture of IBV and MβCD at an MOI of 1 for 2 h at 37 °C, and the cells were collected after 48 h of maintenance culture for detection of viral replication as described above.

To verify whether the effect of cholesterol depletion was reversible, exogenous cholesterol was used to replenish the viral envelope. IBV was treated with 10 mM/mL MβCD for 1 h at 37 °C, and then the supernatant was suspended in medium containing exogenous cholesterol (0.25, 0.5, and 1.0 mg/mL) and incubated for 1 h at 37 °C. After Vero cells were infected with IBV (MOI = 1.0), the virus replication capability was determined.

### RNA-seq analysis of IBV-infected HD11 cells

HD11 cells were seeded into a 10 mm cell culture dish and infected with IBV (MOI = 1.0) for 48 h. Then, the cells were collected for RNA-seq analysis. For the RNA-seq data of IBV infection, the expression of target genes in infected cells relative to uninfected controls was calculated, and log_2_ (fold change) values were used to generate a heatmap. The differentially expressed antiviral genes were subsequently verified via qRT-PCR.

### Analysis of the effects of viperin overexpression on cholesterol levels and IBV replication in Vero cells

Expression plasmids for chicken viperin (NM_001318443.1) were constructed by PCR amplification of cDNA from IBV-infected HD11 cells and were subsequently cloned and inserted into the pEGFP-N1 vector via the primers 5′-ctaccggactcagatCTCGAGgccaccATGCTGCTGGGCGTTCTG-3′ and 5′-ccgcggtaccgtcgaCTGCAGCCAGTCCAGAATCATGTCTGCTTT-3′ to generate the GFP-tagged expression plasmid pEGFP-N1-viperin. Then, Vero cells cultured as monolayers (approximately 70-80% confluent) in a 6-well plate were transfected with the eukaryotic expression plasmid pEGFP-N1-viperin or pEGFP-N1 (as a control) using Lipo8000 Transfection Reagent (Beyotime, Beijing, China) according to the manufacturer’s instructions. GFP fluorescence intensity and luciferase activity were detected at different time points. The cell nuclei were stained with DAPI (Beyotime Biotech, Beijing, China). Identification of viperin expression by Western blotting and qRT-PCR. Subsequently, Vero cells transfected with the plasmid pEGFP-N1-viperin were infected with the IBV Beaudette strain (MOI = 0.1) and cultured at 37 ℃ in 5% CO_2_ for 12, 24, 48, and 72 h; empty pEGFP-N1-transfected cells were used as controls, followed by the determination of IBV replication and total cholesterol levels.

### Analysis of the effects of viperin knockdown on cholesterol levels and IBV replication in Vero cells

The siRNAs targeting viperin and negative control (NC) siRNAs were designed and synthesized by Hanbio Biotechnology (Shanghai, China), and the sequences used were as follows: siViperin (5′-UCUUCCUCAACAUUAAAUCTT-3′, 5′-GAUUUAAUGUUGAGGAAGATT-3′) and siNC (5′-ACGUGACACGUUCGGAGAATT-3′, 5′-UUCUCCGAACGUGUCACGUTT-3′). The siRNAs were transfected into Vero cells using Lipo8000 Transfection Reagent according to the manufacturer’s instructions. The mRNA level of viperin in the siViperin-transfected and NC-transfected cells was tested via qRT-PCR to evaluate the silencing efficiency before the subsequent assays were performed. The transfected cells were subsequently infected with IBV (MOI = 0.1), followed by the determination of IBV replication and total cholesterol levels.

### Analysis of the effect of treatment of viperin-transfected Vero cells with exogenous cholesterol on IBV replication

To explore whether exogenous cholesterol supplementation can rescue viral replication in viperin-transfected cells, Vero cells transfected with viperin for 48 h were infected with IBV (MOI = 0.1) and cultured in DMEM containing 2% FBS plus 1.0 mg/mL cholesterol at 37 °C for 12–72 h. Vero cells were then collected to test the level of IBV replication.

### Statistical analysis

All the data are presented as the means ± standard deviations (SD) and were analysed via GraphPad Prism 8 software. Statistical analysis was performed via one-way analysis of variance (ANOVA) via SPSS 24.0 software. Post hoc analyses were performed via Tukey’s test to identify significant differences. Significant differences are indicated with asterisks (*, *P* < 0.05; **, *P* < 0.01). Different superscript letters indicate significant differences (*P* < 0.05).

## Results

### IBV infection increases the level of cholesterol in Vero cells

**Figure 1:**
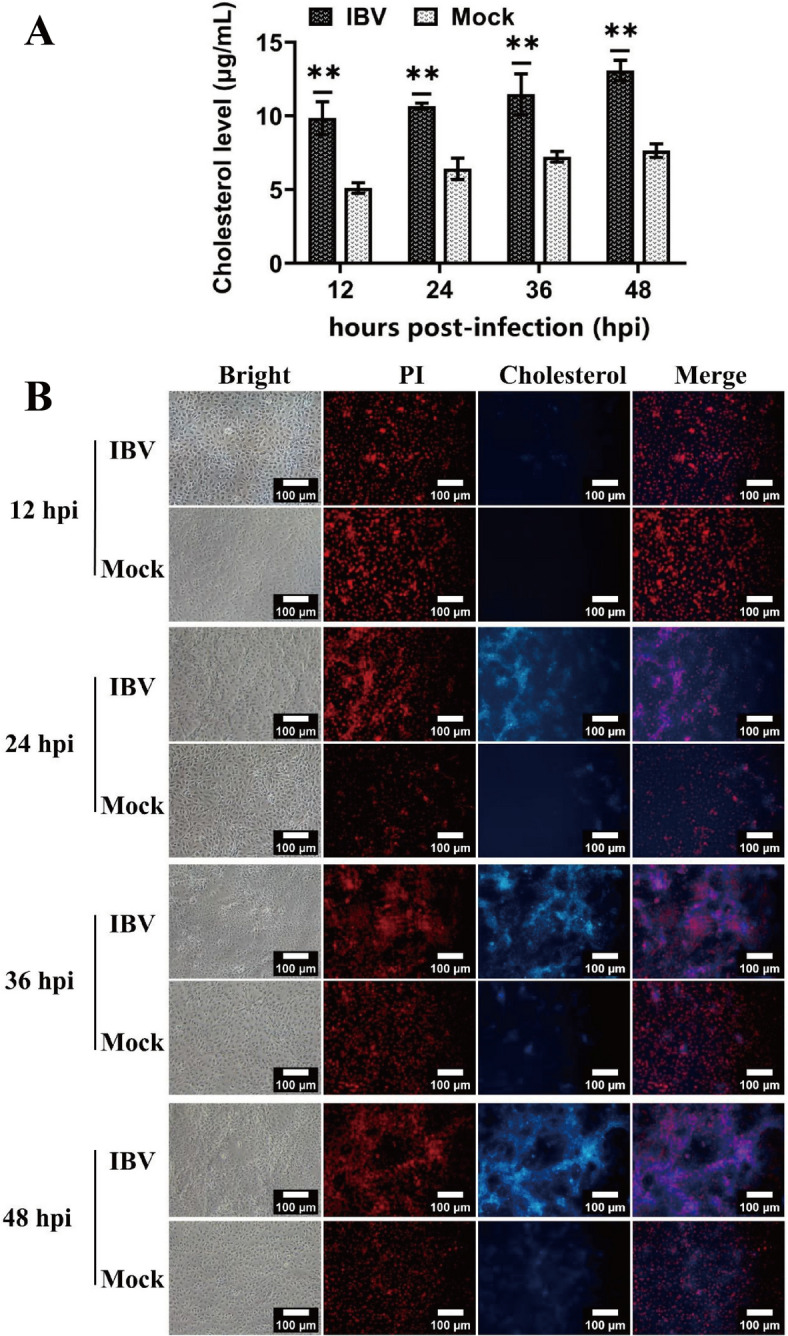
**Cellular cholesterol is upregulated in IBV-infected Vero cells.** **A** Cholesterol levels were quantified with the Amplex™ Red Cholesterol Assay Kit at the indicated time points after infection. **B** Vero cells were fixed with the cholesterol dye filipin (blue), and the nuclei were counterstained with propidium iodide (red) to observe cholesterol enrichment. Bar, 100 μm. **, *P* < 0.01.

To investigate the effect of IBV infection on cellular cholesterol levels, Vero cells were infected with IBV. Compared with those of the mock group, the cholesterol contents of IBV-infected cells were significantly increased as infection progressed from 12 to 48 hpi (Figure 1A). In addition, the accumulation of intracellular cholesterol was observed via filipin III staining, and IBV infection markedly increased intracellular cholesterol accumulation, particularly at 36 and 48 hpi (Figure 1B).

### Cell membrane cholesterol depletion inhibits IBV entry into Vero cells

**Figure 2:**
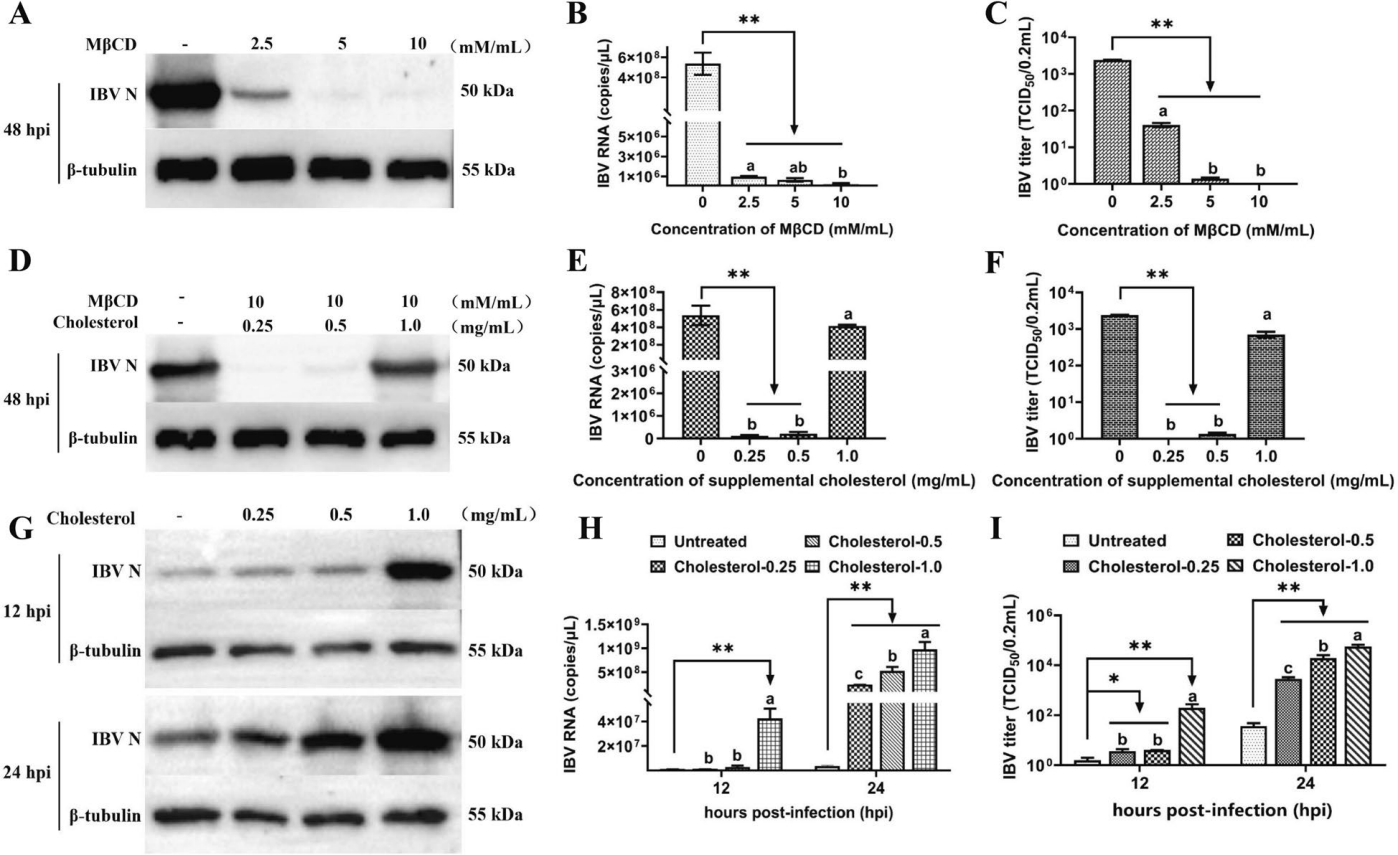
**Effects of cellular cholesterol depletion and replenishment on the replication of IBV in Vero cells. A–C** IBV infection efficiency after cholesterol depletion from the cell membrane. Vero cells were preincubated with MβCD (at various concentrations) or without (−) MβCD and infected with IBV in the presence or absence of MβCD as indicated. The cells and supernatants were collected at 48 hpi to determine **A** N protein, **B** mRNA expression, and **C** supernatant virus titres. **D–F** IBV infection efficiency after cholesterol depletion and replenishment from the cell membrane. Vero cells were preincubated with MβCD and exogenous cholesterol (at various concentrations) or without (−) MβCD and exogenous cholesterol and infected with IBV in the presence or absence of MβCD and exogenous cholesterol as indicated. The cells and supernatants were collected at 48 hpi to determine **D** N protein, **E** mRNA expression, and **F** supernatant virus titres. **G–I** IBV infection efficiency after cholesterol replenishment from the cell membrane. Vero cells were preincubated with exogenous cholesterol (at various concentrations) or without (−) exogenous cholesterol and infected with IBV in the presence or absence of exogenous cholesterol as indicated. The cells and supernatants were collected at 12 and 24 hpi to determine **G** N protein, **H** mRNA expression, and **I** supernatant virus titres. *P < 0.05; **P < 0.01. Different superscript letters indicate significant differences (*P* < 0.05).

To examine whether the depletion of cellular cholesterol affects IBV propagation, Vero cells were pretreated with increasing MβCD concentrations and infected with IBV. The results revealed that virus production was significantly suppressed in MβCD-treated cells in a dose-dependent manner compared with that in untreated cells at 48 hpi (Figures 2A–C). Compared with treatment with 2.5 or 5 mM/mL MβCD, treatment with 10 mM/mL MβCD efficiently reduced the levels of viral N protein (Figure 2A), viral mRNA (Figure 2B), and viral titres (Figure 2C) (*P* < 0.05). Thus, 10 mM/mL MβCD was considered an appropriate concentration with efficient cholesterol depletion for use in subsequent experiments.

To confirm that the inhibitory effects on IBV entry in Vero cells were due to cholesterol depletion, exogenous cholesterol was subsequently used to replenish cholesterol in the cell membrane. The cells were treated or mock-treated with MβCD (10 mM/mL), different concentrations of cholesterol were used for replenishment, and virus reproduction was determined. At 48 hpi, the inhibitory effect of MβCD on IBV entry was reversed by cholesterol supplementation with 1.0 mg/mL exogenous cholesterol (Figures 2D–F), and the difference was not significant (*P* > 0.05) compared with the untreated group. However, the effects of IBV suppression were largely unrecovered with the addition of cholesterol at concentrations of 0.25 and 0.5 mg/mL. There was no significant effect on the expression level of the IBV N protein at 0.25 and 0.5 mg/mL, whereas IBV replication was significantly increased at 1.0 mg/mL (Figure 2D). The viral mRNA levels (Figure 2E) and viral titres (Figure 2F) were consistent with those of the IBV N protein (Figure 2D). These results clearly indicate that a reduction in cellular cholesterol levels leads to a reduction in IBV replication capacity and that viral replication is restored to some extent with the replenishment of cellular cholesterol.

Furthermore, we also tested the effect of exogenous cholesterol supplementation alone on IBV infection. The results revealed that all the different concentrations of cholesterol increased IBV replication at 12 and 24 hpi (Figures 2G-I). The viral protein levels (Figure 2G), viral mRNA levels (Figure 2H), and viral titres (Figure 2I) in cholesterol-treated cells increased significantly (*P* < 0.05) in a dose-dependent manner at 12 and 24 hpi. In addition, viral protein (Figure 2G) and mRNA levels (Figure 2H) and viral titres (Figure 2I) in the cholesterol replenishment group (0.25, 0.5, and 1.0 mg/mL) were significantly greater (*P* < 0.05 or *P* < 0.01) than those in the untreated group at 12 and 24 hpi.

### Viral envelope membrane cholesterol depletion completely depresses IBV replication in Vero cells

**Figure 3:**
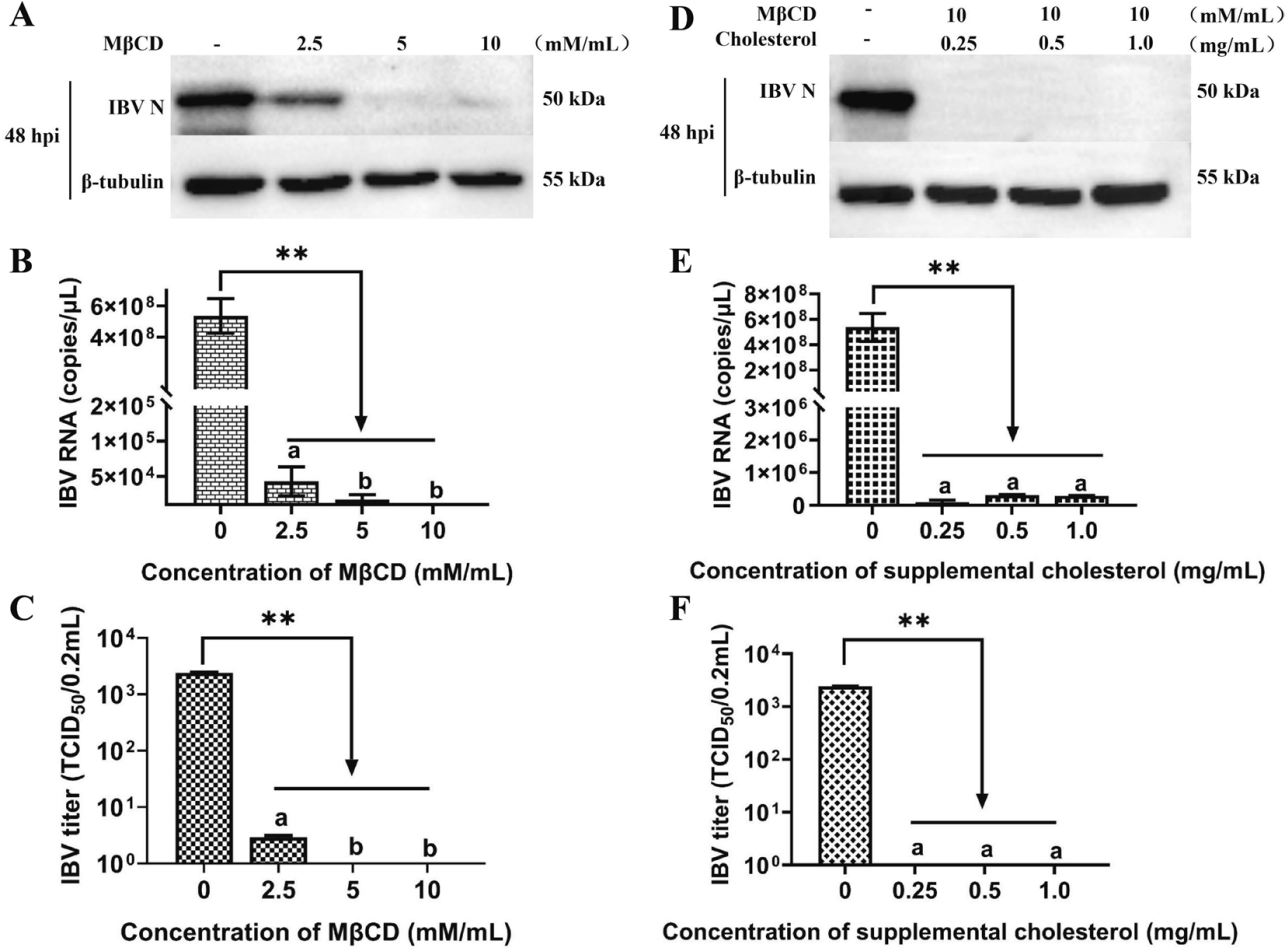
**Effects of viral cholesterol depletion and replenishment on the replication of IBV in Vero cells. A‒C** IBV infection efficiency after cholesterol depletion from the virus envelope. IBV suspensions were treated with MβCD (at various concentrations) or without (−) MβCD, followed by ultracentrifugation and infection. Vero cells and supernatants were collected at 48 hpi to determine **A** N protein, **B** mRNA expression, and **C** supernatant virus titres. **D‒F** IBV infection efficiency after cholesterol depletion and replenishment from the viral envelope. IBV suspensions were treated with MβCD and exogenous cholesterol (at various concentrations) or without (−) MβCD and exogenous cholesterol, followed by ultracentrifugation and infection. The cells and supernatants were collected at 48 hpi to determine **D** N protein, **E** mRNA expression, and **F** supernatant virus titres. **P < 0.01. Different superscript letters indicate significant differences (*P* < 0.05).

To investigate the effect of cholesterol in the viral envelope on IBV replication, the virus stock was treated with MβCD and used to infect Vero cells. The results revealed that the replication of MβCD-treated IBV was significantly impaired compared with that of the untreated stock at 48 hpi (Figures 3A‒C). Consistent with the results of MβCD removal of cell membrane cholesterol, removal of viral envelope membrane cholesterol with different concentrations of MβCD also resulted in significant reductions in viral protein (Figure 3A), viral mRNA (Figure 3B), and viral titre levels (Figure 3C) (*P* < 0.01). However, after cholesterol was replenished, the virus was not restored to a level similar to that in the untreated control (Figures 3D‒F). Thus, supplementation with exogenous cholesterol after the removal of viral vesicular cholesterol did not restore IBV replication.

### IBV infection promotes the expression of viperin and other antiviral genes in HD11 cells

**Figure 4:**
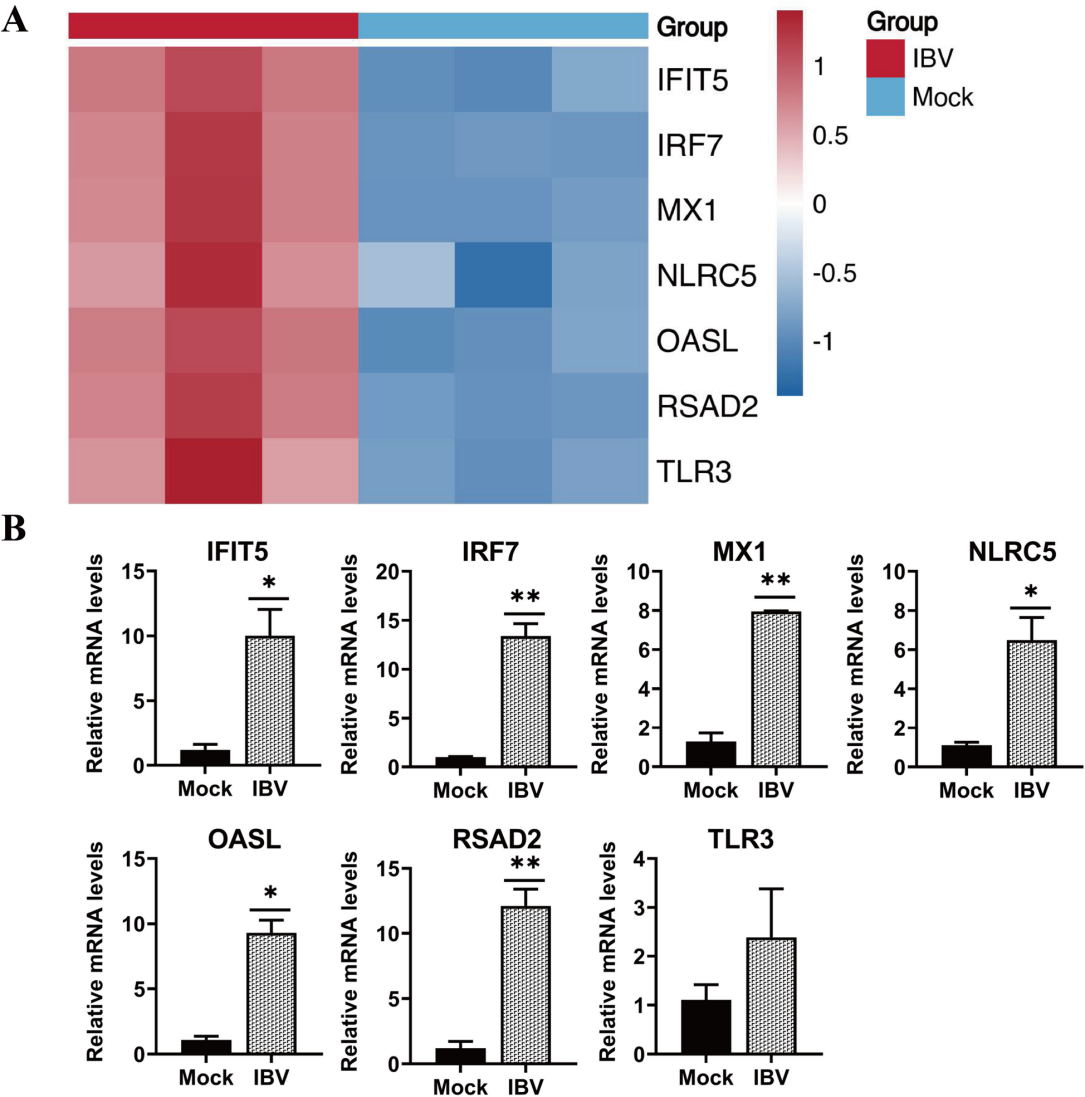
**Viperin is induced by IBV infection in HD11 cells. A** HD11 cells were infected with IBV at an MOI of 1.0 for 48 h for RNA sequencing analysis. The color key represents the log_2_ (fold change) relative to uninfected control cells. **B** Measurement of the expression of IFIT5, IRF7, MX1, NLRC5, OASL, RSAD2, and TLR3 in IBV-infected HD11 cells via qRT-PCR. *, *P* < 0.05; **, *P* < 0.01.

Viperin is an interferon-stimulated gene (ISG) that broadly inhibits viruses, and we sought to investigate the role of viperin in IBV infection. We analysed genes associated with antiviral defense responses induced by IBV infection via RNA-seq data. At 48 hpi, several well-known ISGs, such as IFIT5, IRF7, MX1, NLRC5, OASL, and TLR3, were simultaneously elevated (Figure 4A). Importantly, viperin (RSAD2) expression was significantly upregulated in IBV-infected cells. Similarly, these antiviral genes were verified via qRT-PCR, and the results were consistent with the results of RNA-seq (Figure 4B). Compared with those in the mock group, the expression levels of RSAD2, IFIT5, IRF7, MX1, NLRC5, and OASL in the IBV infection group were markedly increased (*P* < 0.05 or *P* < 0.01) at 48 hpi (Figure 4B). These results suggested that the RNA-seq results were accurate and reliable and that IBV infection promoted viperin expression.

### Viperin overexpression inhibits IBV replication and cholesterol synthesis in Vero cells

**Figure 5:**
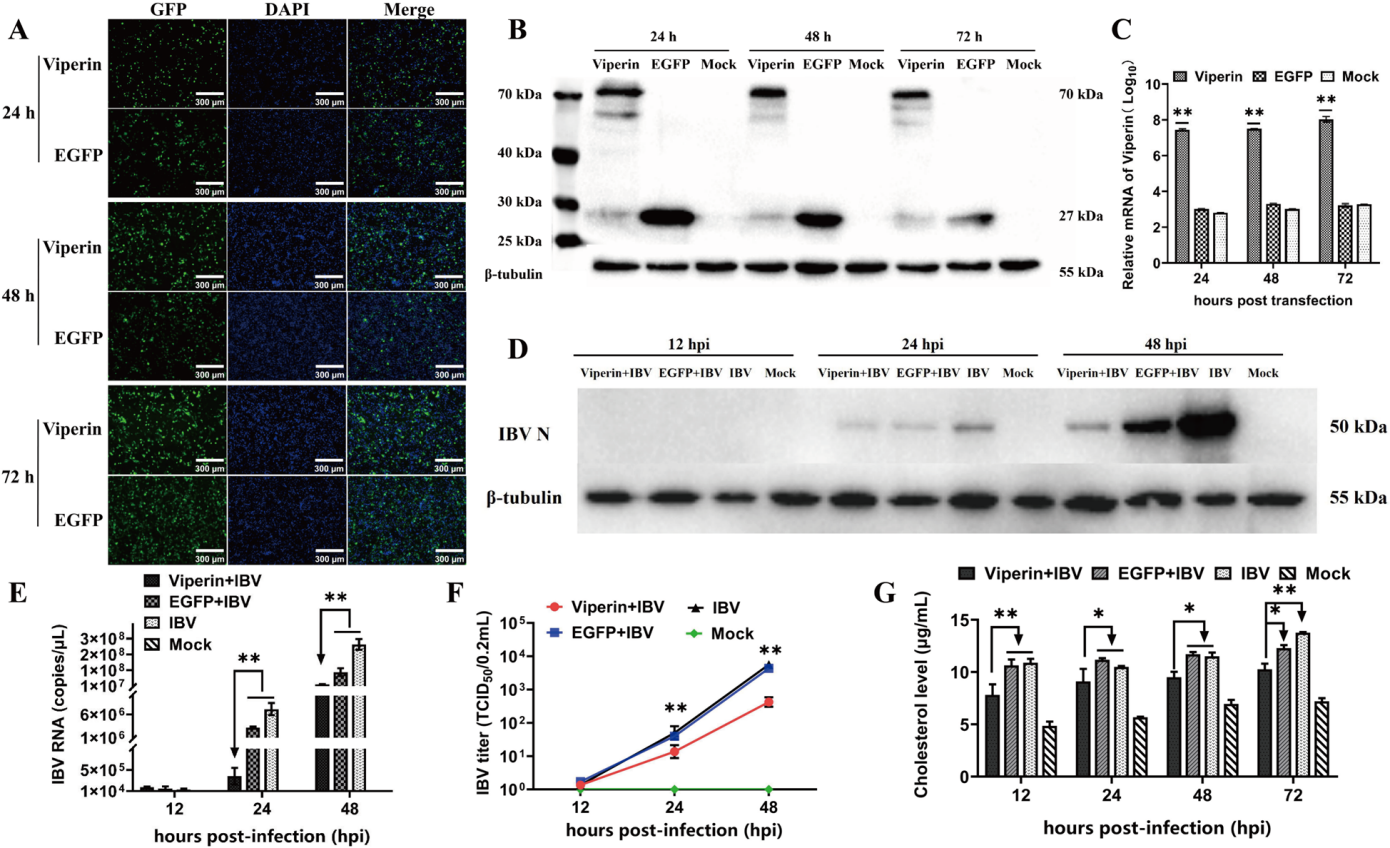
**Overexpression of viperin suppresses IBV replication and cholesterol synthesis in Vero cells. A** Fluorescence microscopy, **B** qRT-PCR or **C** Western blotting verification of the stable expression of viperin in Vero cells. **D‒F** Viperin-overexpressing Vero cells were infected with IBV at an MOI = 0.1. The cells and supernatants were collected at 12, 24, and 48 hpi to determine **D** N protein, **E** mRNA expression, and **F** supernatant virus titres. **G** Cholesterol levels in viperin-overexpressing cells. Bar, 300 μm. *P < 0.05; **P < 0.01.

To verify the overexpression effect of viperin in Vero cells, the eukaryotic expression plasmid pEGFP-N1-viperin with a GFP label was transfected into the cells. The results revealed that at 24–72 h posttransfection, GFP expression could be detected in the viperin-overexpressing cells or the EGFP control cells, and the fluorescence intensity gradually increased with time (Figure 5A). Viperin protein and mRNA levels were assessed by Western blotting (Figure 5B) and qRT-PCR (Figure 5C), and Vero cells successfully overexpressed viperin at 24, 48 and 72 h post-transfection. The levels of viral proteins in cells overexpressing viperin or EGFP were significantly lower than those in IBV-infected cells, and the inhibitory effect of viperin was more pronounced than that of the EGFP control at 24 and 48 hpi (Figure 5D). Consistently, viral mRNA levels (Figure 5E) and viral titres (Figure 5F) were significantly lower in the viperin-overexpressing group than in the infected and EGFP groups (*P* < 0.01) at 24 and 48 hpi, indicating that IBV replication was substantially inhibited by the expression of viperin. In addition, compared with those in the EGFP-transfected and IBV-infected groups, the cholesterol level in the viperin-overexpressing cells significantly decreased (*P* < 0.05 or *P* < 0.01) (Figure 5G) at 12, 24, 48, and 72 hpi, indicating that overexpressing viperin can inhibit cholesterol production.

### Viperin knockdown promotes IBV replication and cholesterol synthesis in Vero cells

**Figure 6:**
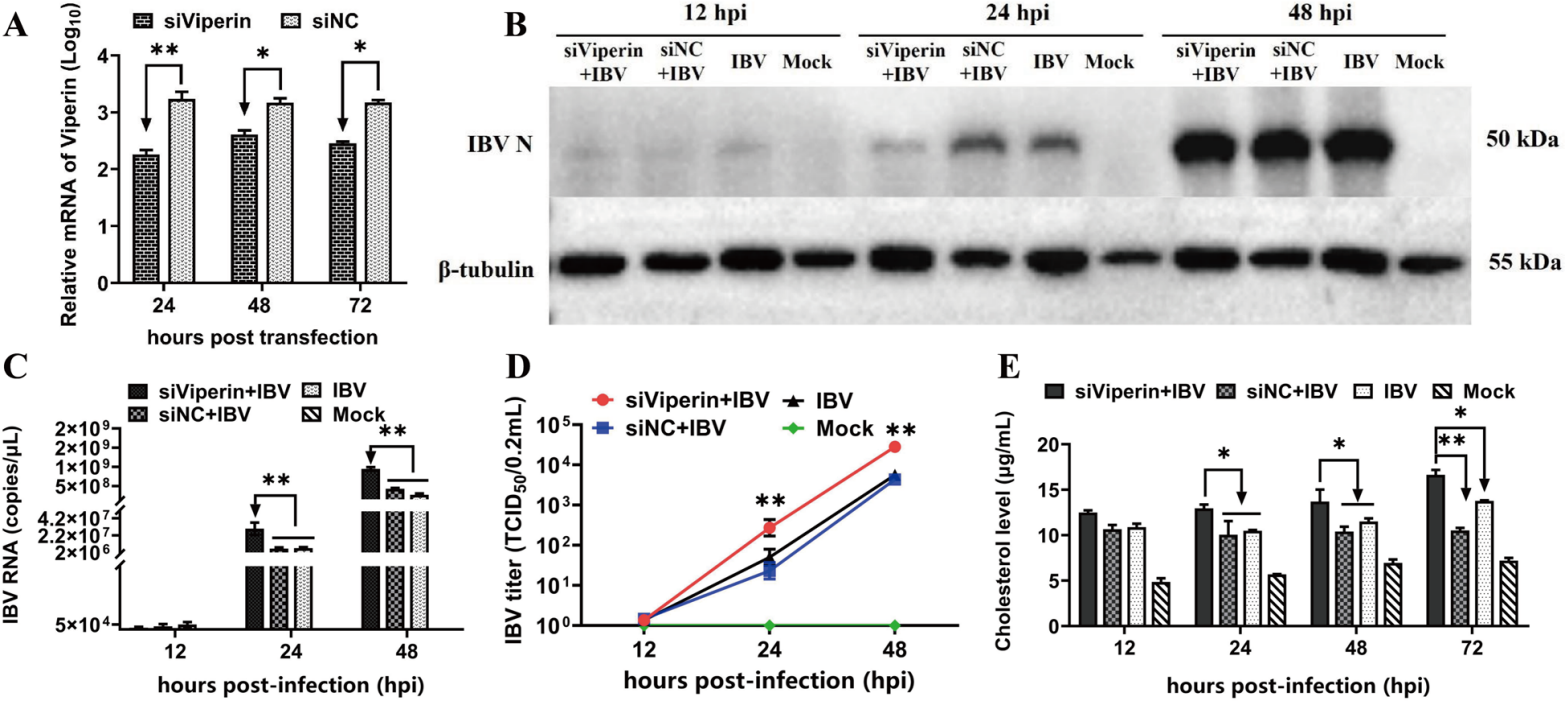
**Viperin knockdown increases IBV infectivity and intracellular cholesterol in Vero cells. A** qRT-PCR validation of the viability knockdown efficiency in Vero cells. **B-D** siViperin-transfected Vero cells were infected with IBV at an MOI = 0.1. Vero cells and supernatants were collected at 12, 24, and 48 hpi to determine **B** N protein, **C** mRNA expression, and **D** supernatant virus titres. **E** Cholesterol levels in siViperin-transfected cells. *P < 0.05; **P < 0.01.

To further assess whether viperin is a host restriction factor against IBV infection, Vero cells were transfected with specific siRNAs targeting chicken viperin to silence the endogenous expression of viperin. Viperin mRNA was assessed by qRT-PCR, and the results demonstrated that treatment with siRNA knocked down viperin expression at 24, 48, and 72 h post-transfection (Figure 6A). Then, siViperin-transfected cells were infected with IBV. Western blotting revealed that IBV N protein levels were not significantly increased in siViperin-expressing cells at 24 and 48 hpi (Figure 6B). Nevertheless, viral mRNA levels (Figure 6C) and viral titres (Figure 6D) in siViperin-expressing cells were significantly greater than those in cells transfected with siNC at 24 and 48 hpi. Thus, on the basis of the results of the overexpression and knockdown assays, we concluded that viperin is a host restriction factor against IBV infection. In addition, we also found that the cholesterol level was significantly greater (*P* < 0.05 or *P* < 0.01) in the viperin knockdown group than in the siNC and IBV groups at 24, 48 and 72 hpi (Figure 6E), suggesting that knockdown of viperin effectively promoted cholesterol synthesis. These results indicate that viperin may regulate cholesterol production to further affect IBV replication.

### Supplementation of viperin-overexpressing vero cells with exogenous cholesterol restores IBV replication

**Figure 7:**
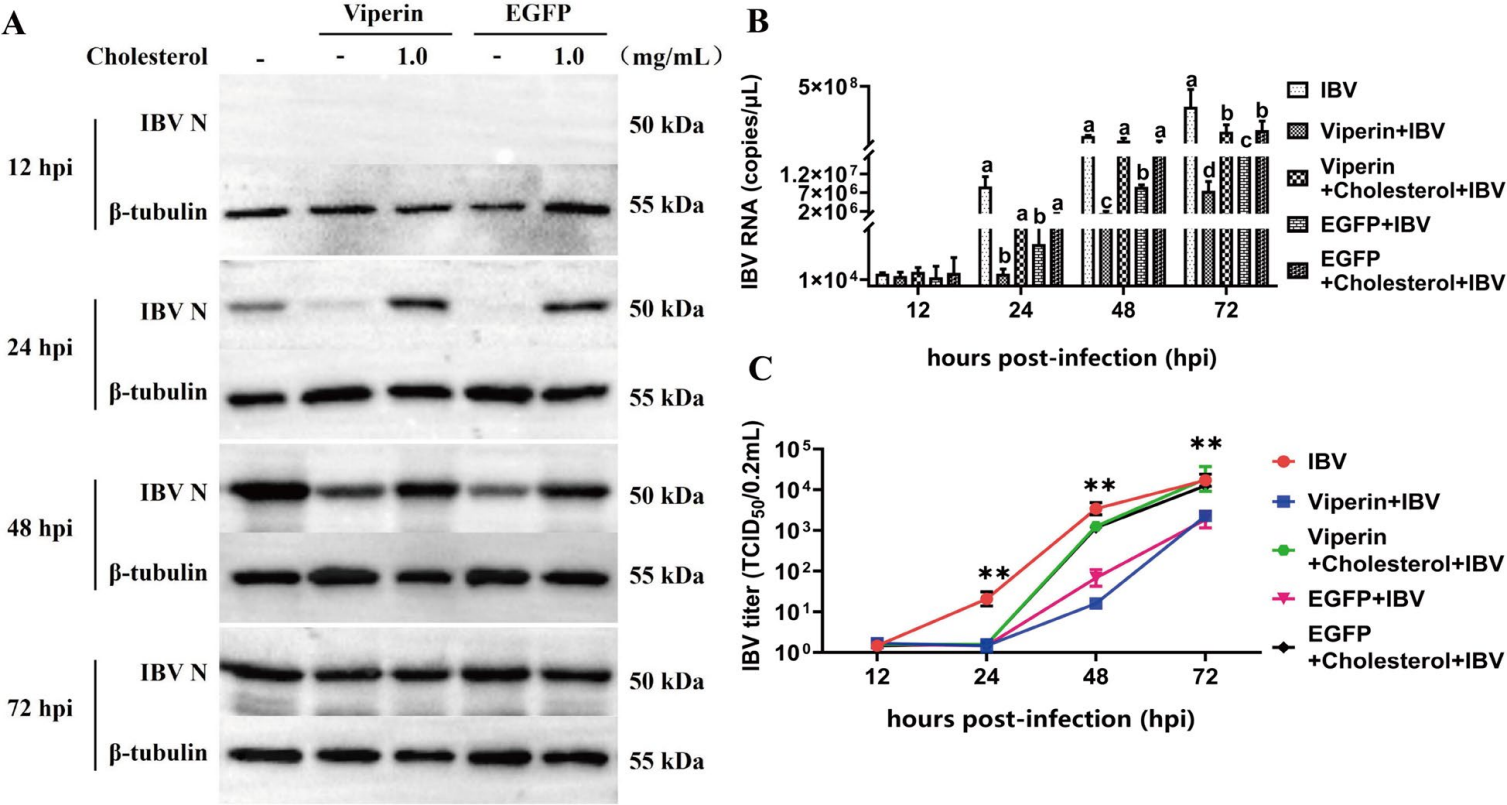
**Effect of supplementation with exogenous cholesterol to viperin-overexpressing Vero cells on IBV replication.** **A‒C** Vero cells overexpressing viperin were preincubated with exogenous cholesterol (at various concentrations) or without (–) exogenous cholesterol and infected with IBV in the presence or absence of exogenous cholesterol as indicated. Vero cells and supernatants were collected at 12, 24, 48, and 72 hpi to determine **A** N protein, **B** mRNA expression, and **C** supernatant virus titres. **P < 0.01. Different superscript letters indicate significant differences (*P* < 0.05).

The above experiments revealed that overexpression of viperin inhibits cholesterol synthesis. We hypothesized that the decrease in cell membrane cholesterol levels triggered by the overexpression of viperin could be responsible for the reduction in viral propagation. To test this possibility, Vero cells were transiently transfected with viperin, followed by the addition of exogenous cholesterol to supplement the cholesterol in the cell membrane. Supplementation of viperin-overexpressing cells with exogenous cholesterol dramatically rescued the replication capacity of IBV (Figures 7A–C), suggesting that viperin can regulate cholesterol production to further affect IBV replication. Indeed, at 24, 48, and 72 hpi, cholesterol replenishment completely restored viral protein levels (Figure 7A), mRNA replication (Figure 7B), and viral titres (Figure 7C) in viperin-overexpressing cells, further supporting a role for viperin in modulating cholesterol-dependent steps during viral replication.

## Discussion

IBV has become an important pathogen that causes severe economic losses in the poultry industry worldwide. The mechanism by which IBV infects host cells is not fully understood, and few virus/host-related studies have been conducted. Thus, it is important to perform virus‒host studies to understand the pathogenic mechanism of IBV. This study is the first characterization of how viperin inhibits IBV replication by restraining cholesterol synthesis, which is highly important for further understanding the pathogenesis of IBV and designing antiviral targets.

Cholesterol is an important component of lipid rafts that is essential for normal cellular physiological functions and may affect viral entry by altering the interaction of viral particles with host cell membranes [12, 37]. In addition, cholesterol plays an important role in the viral life cycle, and many coronaviruses require cholesterol on the viral envelope or cell membrane to enter the cell [11, 25]. Therefore, an in-depth exploration of cholesterol anabolic pathways can help elucidate the mechanisms of virus-host interactions. It has been shown that viruses regulate intracellular cholesterol levels primarily by reducing cholesterol efflux or promoting cholesterol synthesis from scratch [38, 39]. For example, dengue virus (DENV) may prevent cholesterol outflow by reducing low-density lipoprotein receptor-associated protein 1 (LRP-1) protein expression, thereby increasing intracellular cholesterol and promoting viral replication [38]. Porcine reproductive and respiratory syndrome virus (PRRSV) infection activates the enzyme 3-hydroxy-3-methylglutaryl coenzyme A reductase (HMGCR) by reducing protein phosphatase 2 A (PP2A) phosphorylation, causing an increase in de novo cholesterol synthesis, which leads to an increase in cellular cholesterol [39]. In this study, we found significant upregulation of cholesterol levels and enrichment of cholesterol after IBV infection, which was consistent with the above results. However, it remains unclear whether IBV regulates intracellular cholesterol levels primarily by decreasing cholesterol efflux or by promoting cholesterol synthesis from the beginning. These pathways need to be further investigated.

Cholesterol depletion may directly affect virus-cell fusion. The treatment of porcine testicular cells or virions with MβCD can prevent the entry of PDCoV, the addition of exogenous cholesterol can restore PDCoV infection, and cholesterol in the cell membrane and the virus envelope membrane is required for the entry of PDCoV [20]. The virus envelope has properties similar to those of the envelope membrane. The observation that the virus was suppressed in MβCD-treated cells led us to hypothesize that the replication of IBV might also be affected by perturbations in the lipid content (especially cholesterol) of its envelope. In this study, we used the cholesterol-removing drug MβCD to deplete cholesterol from cell membranes and viral envelope membranes, infected IBV and found that viral replication was significantly inhibited, as were viral titres and the expression levels of viral proteins in an MβCD-dependent manner. To confirm that the reduction in replication was the result of cholesterol depletion, the replenishment of the cell membrane with cholesterol successfully restored viral replication to the same level as that of the untreated virus. Similar results were reported in previous studies of infection by IBV [24], SARS-CoV-2 [14], and Newcastle disease virus (NDV) [40]. However, MβCD treatment of virus particles followed by replenishment of the virus envelope with the same concentration of cholesterol did not restore virus replication, contrary to the findings above. It is likely that the large holes that appear in the viral envelope after depletion of cholesterol disrupt the intact structure of the virus, resulting in an inability to be reversed by supplementation with exogenous cholesterol. Our results are consistent with those reported by Aizaki et al., who reported that pretreatment of viral particles with MβCD (exceeding 10 mg/mL) did not restore hepatitis C virus (HCV) replication caused by cholesterol supplementation [41]. Furthermore, we found that the direct addition of exogenous cholesterol to the cell membrane significantly promoted IBV infection, acting mainly in the early stages of infection (at 12 and 24 hpi). Dai et al. [42] also confirmed that the addition of exogenous cholesterol to DF-1 cells promotes IBV replication, which is consistent with our results. Thus, these data suggest that cholesterol plays a vital role in the invasion and replication of IBV in vitro. However, it is still unclear which key synthetic enzyme in cholesterol synthesis is primarily responsible for IBV replication, and the specific underlying mechanisms need to be further explored. It is not clear whether MβCD consumption of cholesterol in vivo results in reduced infectivity of IBV. Future in vivo studies will be necessary.

Viral entry into host cells is a relatively complex process that begins with recognition and specific binding between the virus and the host cell, which is usually mediated by protein or lipid molecules [8]. The molecular mechanisms of virus-host cell interactions are a prerequisite for targeting host cell components and may lead to the development of effective therapeutic strategies. Antiviral drugs can prevent time-consuming vaccine development and viral resistance due to rapid antigenic mutation [43]. Host cytokine targeting is a promising approach that has recently emerged. Viperin is known to have broad-spectrum antiviral effects. Our data indicated that IBV infection promoted the expression of viperin and other antiviral genes. Furthermore, the present study revealed that overexpressing viperin effectively inhibited IBV replication and cholesterol production. In contrast, siRNA-mediated knockdown of viperin significantly promoted IBV replication and increased the cellular cholesterol level. These data suggest that viperin has an antiviral effect against IBV both in vivo and in vitro. A previous study showed that viperin inhibited HCV replication possibly by binding to the host protein hVAP-33 and interfering with its interaction with the nonstructural protein NS5A [44]. In addition, another study showed that avian IFN regulatory factor 3/7 (IRF3/7) may inhibit the replication of duck tembusu virus (DTMUV) by regulating the expression of IFN-β and viperin and that the DTMUV NS2B protein alone can inhibit the induction effect of the downstream gene viperin in infected cells [45]. Similarly, IBV nonstructural proteins play important roles in viral replication and the modulation of the host immune response and are largely responsible for the pathogenicity of IBV [46, 47]. However, it is not clear which nonstructural protein is associated with the viperin-mediated inhibition of IBV. Nsp3 is the largest nonstructural protein encoded in the IBV genome, and the papain-like protease encoded by NSP3 has strong deubiquitination and deISGylating activities, thereby blocking or delaying the host innate immune response of IBV-infected cells [48]. IBV nsp4 can independently induce membrane rearrangements that provide sites for the assembly and synthesis of viral RNA and protect it from host antiviral immune recognition [49]. Therefore, we plan to explore the specific mechanism of IBV inhibition by viperin in the next step and determine whether viperin exerts its antiviral effect through IBV nsp3/4.

Notably, supplementation with exogenous cholesterol after the overexpression of viperin restored the replication capacity of IBV. Our data suggest that host viperin inhibits IBV replication by restraining cholesterol synthesis. There is some evidence that viperin affects lipid raft formation and the viral life cycle during viral infection by blocking key steps in lipid biosynthesis. For example, Wang et al. [29] demonstrated that viperin inhibits the release of IAV and that the destruction of lipid rafts may be due to the inhibition of FPPS (a key enzyme in cholesterol synthesis) by viperin. Tang et al. [30] reported that viperin could inhibit the replication and release of RABV by inhibiting viral outgrowth and disrupting cholesterol/sphingolipids on cell membranes. Cholesterol production was significantly upregulated in the absence of viperin during viral hemorrhagic septicemia virus (VHSV) infection in zebrafish larvae, and overexpression of viperin significantly reduced lipid production and significantly upregulated reactive oxygen species (ROS) production [50]. Moreover, human immunodeficiency virus (HIV) infection is induced by viperin to destroy lipid rafts and redistribute viperin to the CD81 compartment (the exit site of HIV in macrophage germination), and the addition of exogenous farnesol reverses viperin-mediated HIV inhibition [51]. Therefore, we hypothesize that the inhibition of IBV replication by viperin may inhibit IBV outgrowth by inhibiting cholesterol synthesis, thereby reducing membrane fluidity and disrupting lipid rafts at the envelope membrane. Future studies should focus on how IBV specifically utilizes cholesterol to enter target cells and examine the relationship between viperin and key enzymes of cholesterol synthesis, in addition to the possibility that other components of lipid rafts, such as sphingolipids, contribute to this process, thereby elucidating the exact mechanism by which cholesterol-rich lipid rafts mediate IBV entry. This information is conducive to a better understanding of virus‒host interactions in the early stages of viral infection.

In conclusion, our data suggest that cholesterol in both the cellular and viral envelope membranes plays an important role in IBV replication and suggest a possible mechanism by which viperin inhibits IBV replication by inhibiting cholesterol synthesis. Valuable insights into the antiviral mechanism of viperin are provided and may be used in the development of novel antiviral drugs for the treatment of IBV.

## Data Availability

The data that support the findings of this study are available from the authors upon reasonable request.
